# Epigenetic regulation in epithelial cells and innate lymphocyte responses to *S*. Typhi infection: insights into IFN-γ production and intestinal immunity

**DOI:** 10.3389/fimmu.2024.1448717

**Published:** 2024-09-20

**Authors:** Rosângela Salerno-Goncalves, Haiyan Chen, Andrea C. Bafford, Marcelo B. Sztein

**Affiliations:** ^1^ Center for Vaccine Development and Global Health and Department of Pediatrics, University of Maryland School of Medicine, Baltimore, MD, United States; ^2^ Division of General and Oncologic Surgery, University of Maryland School of Medicine, Baltimore, MD, United States; ^3^ Program in Oncology, University of Maryland Marlene and Stewart Greenebaum Comprehensive Cancer Center, Baltimore, MD, United States

**Keywords:** bacteria, salmonella, epigenetic, human, gut

## Abstract

Infection by *Salmonella enterica* serovar Typhi (*S*. Typhi), the cause of enteric fevers, is low in high-income countries but persistent in low- and middle-income countries, resulting in 65,400-187,700 deaths yearly. Drug resistance, including in the United States, exacerbates this issue. Evidence indicates that innate lymphocytes (INLs), such as natural killer (NK) cells, and unconventional T lymphocytes (*e.g*., Mucosal-associated invariant T (MAIT) cells and T-cell receptor gamma delta (TCR-γδ) cells) can impact the intestinal epithelial barrier, the primary site of exposure to *S*. Typhi. Moreover, INL production of IFN-γ is central in controlling *S*. Typhi infection. However, the impact of epithelial cells (EC) on the secretion of IFN-γ by INLs and the relationship between these events and epigenetic changes remains unknown. Epigenetic modifications in host cells are fundamental for their differentiation and function, including IFN-γ production. Herein, using a human organoid-derived polarized intestinal epithelial cell monolayer, we investigated the role of H3K4me3 and H3K27me3 epigenetic marks in intestinal immunity, focusing on the function of EC, NK, MAIT, and TCR-γδ cells in response to *S*. Typhi. This study builds on our previous findings that MAIT subsets exhibiting specific IFN-γ pattern signatures were associated with protection against typhoid fever and that *S*. Typhi infection regulates changes in chromatin marks that depend on individual cell subsets. Here, we show that cultures exposed to *S*. Typhi without EC exhibit a significant increase in NK and MAIT cells, and, to a lesser extent, TCR-γδ cells, expressing IFN-γ and H3K4me3 but not H3K27me3 marks, contrasting with cultures where EC is present. The influence of EC on INL H3K4me3 marks might be indirectly mediated through the modulation of IL-18 secretion via the Histone Deacetylase 6 gene during *S*. Typhi infection.

## Introduction

The incidence of enteric fevers remains notably low in industrialized countries. In the United States, approximately 5,700 cases are reported each year, with the majority linked to international travel (1). However, in low- and middle-income countries, enteric fevers continue to pose a significant public health challenge, causing an estimated 65,400-187,700 deaths annually (2–6). Furthermore, cases of multi-drug-resistant *Salmonella enterica* serovar Typhi (*S.* Typhi), the pathogen responsible for typhoid fever, have surfaced even in developed countries. In the United States, cases of multidrug-resistant *S*. Typhi have been reported even among individuals without recent international travel history (1). Chronic typhoid carriers and inadequate access to clean water and sanitation also contribute to the persistence of enteric fevers (7, 8).

Growing evidence suggests that innate lymphocytes (INLs) play a significant role in influencing the structure and function of the epithelial barrier in the intestine, which is the primary site of exposure to *S*. Typhi (9, 10). The intestinal immune system has evolved to tolerate most antigens while responding effectively to combat pathogen infections (11). INLs such as intestinal natural killer (NK) cells, and innate T cells (*i.e*., Mucosal-associated invariant T (MAIT) cells and T-cell receptor gamma delta (TCR-γδ) cells) are crucial for initiating and regulating immune responses to pathogens, cytokine production, and tissue repair (9, 10). Innate T cells are unconventional T cells restricted by monomorphic MHC class Ib (MHC-Ib) molecules that seed tissues during development, and recognize highly conserved antigens, suggesting a critical role in maintaining tissue homeostasis (9). Recent evidence shows that these cells form a network with epithelial cells (EC) and have both distinct and overlapping functions (9). Epigenetic modifications play vital roles in regulating the differentiation and function of intestinal EC, NK, and innate T cells (12–14). These modifications can also be influenced by intracellular pathogens (15) and cytokines such as IFN-γ (16–19). Conversely, epigenetic marks can regulate the expression of Th1 genes and the IFN locus (20–24). Our previous studies have shown that MAIT cells displaying distinct cytokine patterns (*i.e.*, IFN-γ^+^ TNF-α^+^ IL-17A^-^ positive cells) were associated with protection against typhoid fever (25). Of note, IFN-γ+ TNF-α+ IL-17A- MAIT cells constitute the majority of IFN-γ+ MAIT cells (~65%), while only a minority of MAIT cells expressing IFN-γ lack TNF-α (~16%) (26). Our previous studies also showed that changes in chromatin marks resulted from *S*. Typhi infection and were dependent on individual cell subsets (15). In addition, our group demonstrated that the crosstalk between lymphocytes and intestinal EC is cytokine/chemokine-dependent, bacterial-serotype specific, and plays a pivotal role in orchestrating the functional efficiency of innate cells (27, 28). However, the impact of gut EC on the secretion of IFN-γ by INLs and the relationship between these events and epigenetic changes remains unknown. Here, we investigated the epigenetic modifications in the IFN-γ expressing INLs, such as NK and innate T cells (*i.e*., MAIT and TCR-γδ cells) after exposure to *S*. Typhi in the presence or absence of intestinal EC. We focused on epigenetic marks, which are known to mediate transcription of the IFN-γ gene (*i.e.*, H3K27me3 and H3K4me) (29, 30). We hypothesized that the modulation of cytokine production (*i.e*., IFN-γ) is achieved, at least in part, by early epigenetic modifications following exposure to *S*. Typhi. Here, we show that cultures exposed to *S*. Typhi without EC exhibit a significant increase in NK and MAIT cells, and, to a lesser extent TCR-γδ cells, expressing IFN-γ and H3K4me3 but not H3K27me3 marks, contrasting with cultures where EC is present. The influence of EC on INL H3K4me3 marks might be indirectly mediated through the modulation of IL-18 secretion via the Histone Deacetylase 6 gene during *S*. Typhi infection.

## Materials and methods

### Ethics statement

Seven healthy volunteers aged between 24 and 41 from the Baltimore-Washington area were recruited for this study. They underwent screening for typhoid vaccination, general health through medical history and examination, and normal laboratory tests, including blood cell counts. They were also checked for the absence of recent antibiotic treatment. The volunteers were informed about the study’s purpose and provided signed consent. Peripheral blood mononuclear cells (PBMC) were isolated from their blood using density gradient centrifugation and stored in liquid nitrogen for later use. The study adhered to the human experimentation guidelines of the US Department of Health and Human Services and the University of Maryland, Baltimore. Blood specimens were collected under the University of Maryland Institutional Review Board-approved protocol HP-00040025.

Anonymized residual terminal ileum tissues from seven adults were collected from patients undergoing surgery as per standard of care. The tissues were determined to be normal based on a macroscopic inspection by the surgeon. Specimens were de-identified such that the subject’s identity was unknown and may not readily be ascertained by the investigator or any other individual associated with the investigation, including the sponsor. A protocol describing the collection and use of these samples has been submitted to the University of Maryland Institutional Review Board (IRB), and a study exemption has been approved (#HP-00077485). These tissues were used to isolate cells (15), and harvest intestinal crypt-containing stem cells (SC) (31) as previously described

### Preparation of Human organoid-derived polarized intestinal epithelial cell monolayer model

**Figure 1:**
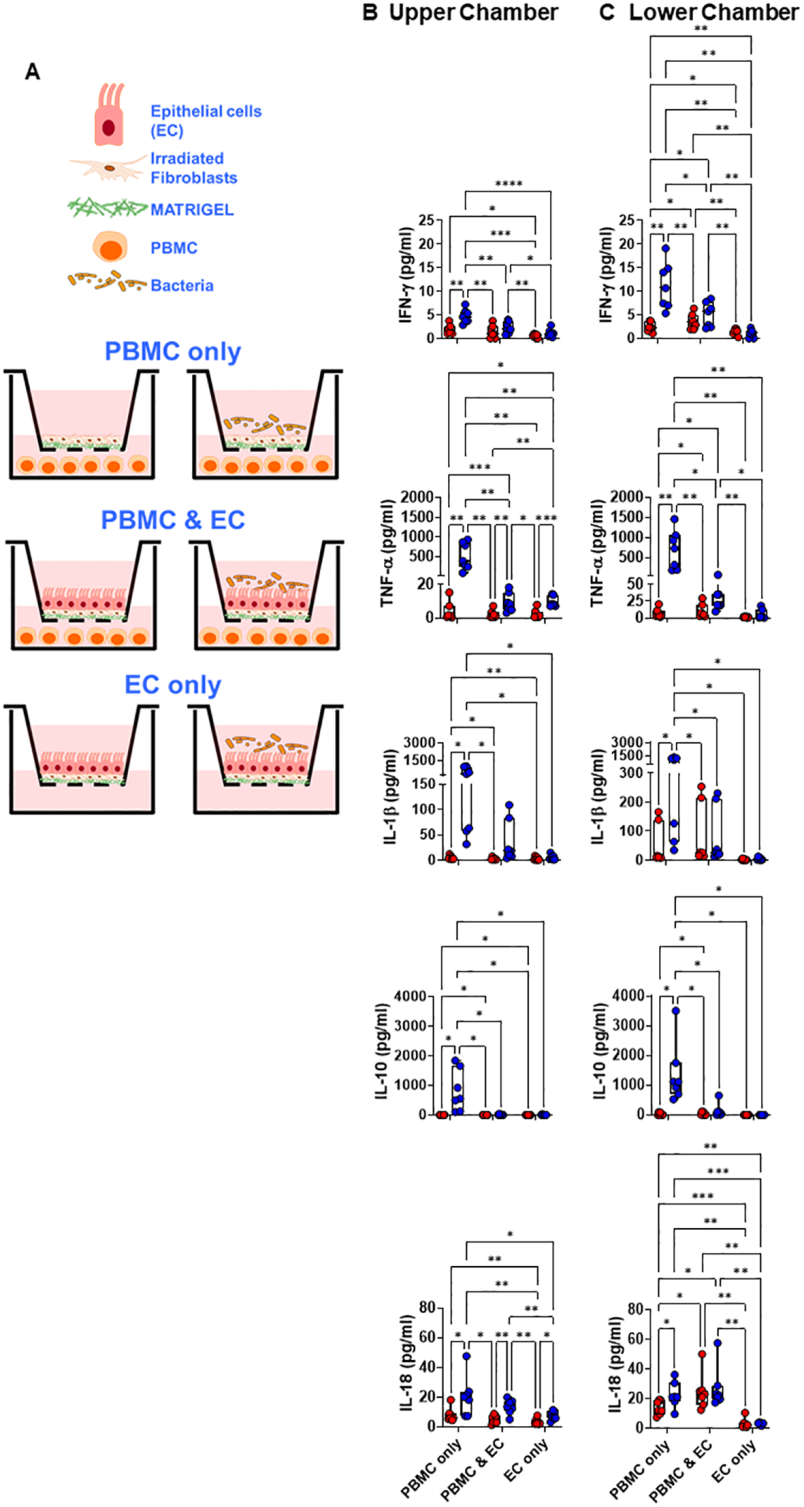
Crosstalk between migrating immune cells and intestinal epithelial cells and the consequences for host responses to *S*. Typhi. **(A)** Cartoon of the experimental design. **(A-C)** Cultures were left untreated (⬤, media only) or exposed to *S*. Typhi strain Ty2 (⬤) in the presence (PBMC only, or PBMC & EC [epithelial cells]) or absence of PBMC (EC only). After 16 hours, supernatants were harvested and used to determine cytokine/chemokine secretion in the upper **(B)** and lower compartment **(C)**. Mixed-effects models were used to compare groups. Data are representative of 7 independent experiments. *P* values < 0.05 were considered statistically significant. The levels of significance are: *, 0.01 to 0.05; **, 0.001 to 0.01; ***, 0.0001 to 0.001; ****<0.0001.

Isolation of terminal ileum SC, culture, and establishment of the HODIM model were performed as previously described (28, 31) (**Figure 1A**). Briefly, SC were co-cultured with irradiated fibroblasts in 6-well-plates coated with Matrigel (Becton Dickinson, New Jersey, USA) and exposed to a cocktail of growth factors. Once the SCs attained adequate numbers for experimentation, colonies of undifferentiated SCs were dissociated and plated onto inserts (upper chamber) of a transwell plate (Corning Inc, Corning, NY, USA) for differentiation into epithelial cells. After up to 5 days of differentiation, PBMC were added to the lower chamber of the HODIM (**Figure 1A**). The apical portion of the epithelial cells in the upper chamber of the HODIM were exposed to *S*. Typhi (**Figure 1A**). In some experiments, cells exposed to *S*. Typhi were treated with the H3K4me3/H3K27me3 demethylase inhibitor GSK-J4 (10μM)(TOCRIS, Minneapolis, MN, USA). It is important to note that each HODIM were built using the stem cells from a single donor.

### Isolation of intestinal cells from healthy adult tissues

Terminal ileum explants were used to isolate intestinal cells (15), as previously described. Briefly, after crypt removal (used for establishing HODIM), and collagenase IV/dispase treatment, cells were filtered through a 100μm mesh, and single-cell suspensions cryopreserved in liquid N_2_ until use. Infection was performed as described (27, 32, 33). Briefly*,* on the day of infection, isolated cells were thawed, incubated for 2 hours on plain DMEM in a 37^0^C 5% CO_2_ incubator, and exposed or not to wild-type *S*. Typhi strain Ty2 at a multiplicity of infection (MOI) of 50:1 for 16 hrs.

### *S.* Typhi infection

The HODIM models were incubated for 6 hours at 37°C in DMEM media (without antibiotics) in the presence of wild-type *S*. Typhi (strain Ty2) (34) at a MOI of 50 (27, 32, 33). HODIM models with media only were used as controls. Following this incubation, the chambers were washed to remove non-attached bacteria, and then subjected to gentamicin treatment to kill attached bacteria. Subsequently, an additional 16-hour culture was carried out before collecting supernatants and cells. Supernatants in the upper and lower chambers were used for cytokine measurements. EC from the upper chamber and PBMC from the lower chamber were collected for mRNA extraction and evaluation of phenotypic and epigenetic changes, respectively.

### Epigenetic landscape profiling by time-of-flight

EpiTOF (15, 35, 36) is a mass cytometry-based analytical platform simultaneously focusing on epigenetic and immunological markers at the single-cell level to create an immune cell epigenetic atlas based on chromatin modification profiles. EpiTOF can concurrently interrogate millions of individual cells for chromatin changes (15). We followed the procedures described previously (15) with small modifications. Briefly, cells were stained for live/dead cells with Cisplatin (198 Pt) (Standard BioTools, San Francisco, CA, USA), followed by human Fc receptor blocking and incubation with a metal-labeled antibody cocktail for surface markers to identify cell lineages (*e.g*., NK, MAITs and TCRγδ cells), and their levels of activation (*i.e.*, CD69). Cells were then fixed and permeabilized, and intracellularly stained with metal-labeled Abs specific to cytokines (*i.e.*, IFN-γ and TNF-α), and histone marks (*i.e*., H3, H3K4me3, H3K27me3) (**Supplementary Table 1**). β2 and CD298 markers were used to barcode the cells so that cells from the different culture conditions could be acquired simultaneously in the same tube to ensure that there is no variation in sample acquisition among culture conditions. Finally, samples were stained with 103Rh (Standard BioTools) for cell detection, re-suspended in normalization beads, and acquired within 48 hours by mass cytometry. Data acquisition was performed using a Helios mass cytometer (Standard BioTools). Mass cytometry experiments were performed at the Flow Cytometry and Mass Cytometry Core Facility of the University of Maryland School of Medicine Center for Innovative Biomedical Resources (CIBR), Baltimore, Maryland.

### qRT-PCR

RNA was isolated from EC in the upper chamber of the HODIM model following established protocols with minor adjustments (27, 28, 33, 37). Briefly, membranes were excised from the upper chamber and transferred into power ceramic beads tubes (Qiagen, Valencia, CA, USA) containing 1 mL of RNA RLT Buffer (RNeasy Micro kit, Qiagen). Subsequently, they were homogenized using a Bullet Blender Homogenizer for 30 seconds. Total cellular RNA was then extracted according to the manufacturer’s protocol and subjected to cDNA synthesis using the RT2 First Strand Reverse Transcription Kit (Qiagen). For each quantitative reverse transcription PCR (qRT-PCR) reaction, 20-50 ng of cDNA was utilized, with the amplified products detected using RT² SYBR^®^ Green qPCR Mastermix (Qiagen). qRT-PCR was conducted on an ABI 7900HT thermocycler (Applied Biosystems). Following amplification, the PCR product sizes were assessed on a 1.0% agarose gel to estimate amplicon sizes. Data analysis was performed using the web-based GeneGlobe Data Analysis Center software (Qiagen), which automatically selected an optimal set of internal control/housekeeping/normalization genes from the available panel (*i.e*., ACTB, B2M, GAPDH, HPRT1, and RPLP0) on the PCR Array. A set of 168 genes was profiled using two RT² Profiler™ PCR Array (*i.e*., Human Epigenetic Chromatin Modification Enzymes [Cat. # PAHS-085Z] (**Supplementary Table 2**), and Human Anti-bacterial Response [Cat. # PAHS-19 148Z]) (**Supplementary Table 3**),) (Qiagen), 84 genes per array. Internal positive controls were used in each experiment to assess the reproducibility of the PCR, with passing CT values’ score for a sample of 20 ± 2.

### Cytokine/chemokine detection

Production of 10 cytokines/chemokines (*i*.*e*., IFN-γ, IL-1β, IL-6, IL-8, IL-10, IL-18, IL-23, and TNF-α) were measured by LEGENDplex™ Human Inflammation Panel multiplex-assays (BioLegend, San Diego, CA). Samples were processed per the manufacturer’s protocol, and the data was analyzed using LEGENDplex™ Data Analysis Software V8.0 (BioLegend). Production of IL-15 was measured by LEGEND MAX™ ELISA kit (BioLegend). Samples were processed per the manufacturer’s protocol.

### Western blot

Western blots were performed according to previous protocols with few modifications (28, 33). Briefly, after 16 hours of incubation with the *S*. Typhi strain, PBMC from the bottom of the well were harvested, PBS washed, and mixed 1 mL of ready-to-use RIPA buffer (Sigma-Aldrich) supplemented with a cocktail of protease and phosphatase inhibitors (Sigma-Aldrich) to prepare the whole-cell lysates. The mixture was briefly vortexed and incubated on ice for 30 min. The lysates were then clarified by centrifugation at 10,000 x g for 15 min at 4 oC, transferred to new microfuge tubes, and the pellets discarded. The cell lysates’ protein concentration was determined using a Thermo Scientific Pierce Micro BCA Protein Assay kit (Thermo Scientific, Rockford, IL, USA). The following primary anti-human antibodies were used for Western Blot: (1) rabbit anti-H3K27me3 recombinant antibody (1:2000), (2) rabbit anti-H3K4me3 polyclonal antibody (1:250), (3) rat anti-H3.1 mAb (clone 5D10D4) (1:250) and (4) rabbit anti-glyceraldehyde-3-phosphate dehydrogenase (GAPDH) polyclonal antibody (1:8000) (R&D Systems, Minneapolis, MN).

### Statistical analysis

All statistical analyses were conducted using Prism software (version 9, GraphPad Software, La Jolla, CA). Mixed-effects models were employed for comparisons involving multiple groups. All *p* values were two-sided, and when less than 0.05, differences were considered significant.

## Results

### Secretion of cytokines/chemokines after exposure to *S.* Typhi using a human organoid-derived polarized intestinal epithelial cell monolayer and impact of crosstalk between innate T cells and epithelial cells

First, we investigated the impact of *S.* Typhi infection in producing 8 cytokines/chemokines, including IL-18, known to induce NK, MAIT, and TCR-γδ cells to produce IFN-γ (38–40). To this end, we used the HODIM to model the *in vivo* environment of the human intestine (31). This experimental setup involved a 6-hour upper chamber *S*. Typhi exposure, followed by chamber washing, gentamicin treatment to remove bacteria that were not cell attached and an additional 16-hour culture. We conducted experiments with (present) or without (absent) peripheral blood mononuclear cells (PBMC) in the HODIM lower chamber (**Figure 1A**). PBMC contains lymphocytes such as INLs (*e.g.*, NK, MAIT, and TCR-γδ cells), and were used to simulate the impact of immune cells migrating to the gut. Immune cells such as INLs recirculate through peripheral blood into different tissues, including the intestinal mucosa (41, 42). HODIM cultured with media only (**Figure 1A**, left 3 panels), without PBMC (**Figure 1A**, EC only, 2 bottom panels), or with PBMC without EC (**Figure 1A**, PBMC only, 2 top panels) were used as controls. Culture supernatants were collected from the upper and lower chambers after the final 16 hours of incubation to measure cytokine/chemokine secretion.

For supernatants collected from the upper chamber of PBMC & EC co-cultures (**Figure 1A**, 2 middle panels, PBMC & EC), after exposure to *S.* Typhi, secretion of TNF-α, IL-6, IL-18, IL-23 significantly increased compared to controls with media-only (**Figure 1B** and **Supplementary Figure 1A**). However, no significant increases were observed for IFN-γ, IL-1β, IL-8, and IL-10 cytokines/chemokines when comparing cultures exposed to *S*. Typhi with those in media only (**Figure 1B** and **Supplementary Figure 1A**). Excepting IFN-γ and regardless of the presence of *S*. Typhi, their levels were similar to cultures with EC only (**Figure 1B** and **Supplementary Figure 1B**). Interestingly, IFN-γ levels were significantly lower in PBMC & EC co-cultures compared to PBMC-only cultures but were significantly higher than in EC-only cultures (**Figure 1B**). It is important to note that EC do not produce IFN-γ (43–45). Thus, these results indicate that while most cytokines/chemokines in the upper chamber originate from EC and PBMC which cannot traverse the 0.4 μm membrane (*i.e.*, small lymphocytes have an approximate size of 6–12 μm (46)), a portion of these cytokines/chemokines are produced by PBMC in the lower chamber. In addition, the significant increase in TNF-α, IL-18, IL-6, and IL-23 levels observed in co-cultures of PBMC and EC cultures exposed to *S*. Typhi compared to controls with only media suggest that the incubation period post-*S*. Typhi infection was sufficient for signaling between ECs and PBMC. Furthermore, the fact that IL-18 levels post-*S*. Typhi infection were comparable between PBMC & EC co-cultures and PBMC-only cultures, without any additional increase from EC-only setups, suggests that PBMCs may influence the EC production of IL-18 after *S*. Typhi exposure (**Figure 1B**). Notably, in PBMC-only culture controls, the levels of all cytokines/chemokines were consistently and significantly elevated when exposed to *S*. Typhi compared to media-only conditions (**Figure 1B** and **Supplementary Figure 1A**). This demonstrates the ability of PBMC to produce all the pre-selected cytokines/chemokines following *S*. Typhi exposure.

Supernatants collected from the lower chamber of PBMC & EC co-cultures (**Figure 1A**, 2 middle panels, PBMC & EC) exposed to *S*. Typhi did not exhibit increases in the secretion of cytokines/chemokines compared to media-only controls (**Figure 1C** and **Supplementary Figure 1B**). However, significant increases were observed for IFN-γ, TNF-α, and IL-18 when comparing PBMC & EC co-cultures with EC-only cultures exposed to *S.* Typhi (**Figure 1C** and **Supplementary Figure 1B**). Similar to supernatants from the upper chambers, in PBMC-only cultures, supernatants from the lower chambers exhibited consistently and significantly higher levels of all cytokines/chemokines when exposed to *S*. Typhi compared to media-only conditions (**Figure 1C** and **Supplementary Figure 1B**). Finally, significant decreases were observed in IFN-γ, TNF-α, IL-10, and IL-23 levels when comparing PBMC & EC co-cultures with PBMC-only cultures, regardless of *S*. Typhi exposure. These findings imply a pivotal role of EC in suppressing the secretion of IFN-γ, TNF-α, IL-10, and IL-23 by PBMC, independent of exposure to *S.* Typhi. These findings support the hypothesis of crosstalk between PBMC and EC, highlighting the utility of PBMC within the HODIM to simulate the activation and epigenetic changes in INLs migrating towards the gut.

### Epithelial cell-immune cell crosstalk and the impact on chromatin-modifying enzymes and anti-bacterial responses of epithelial cells

**Figure 2:**
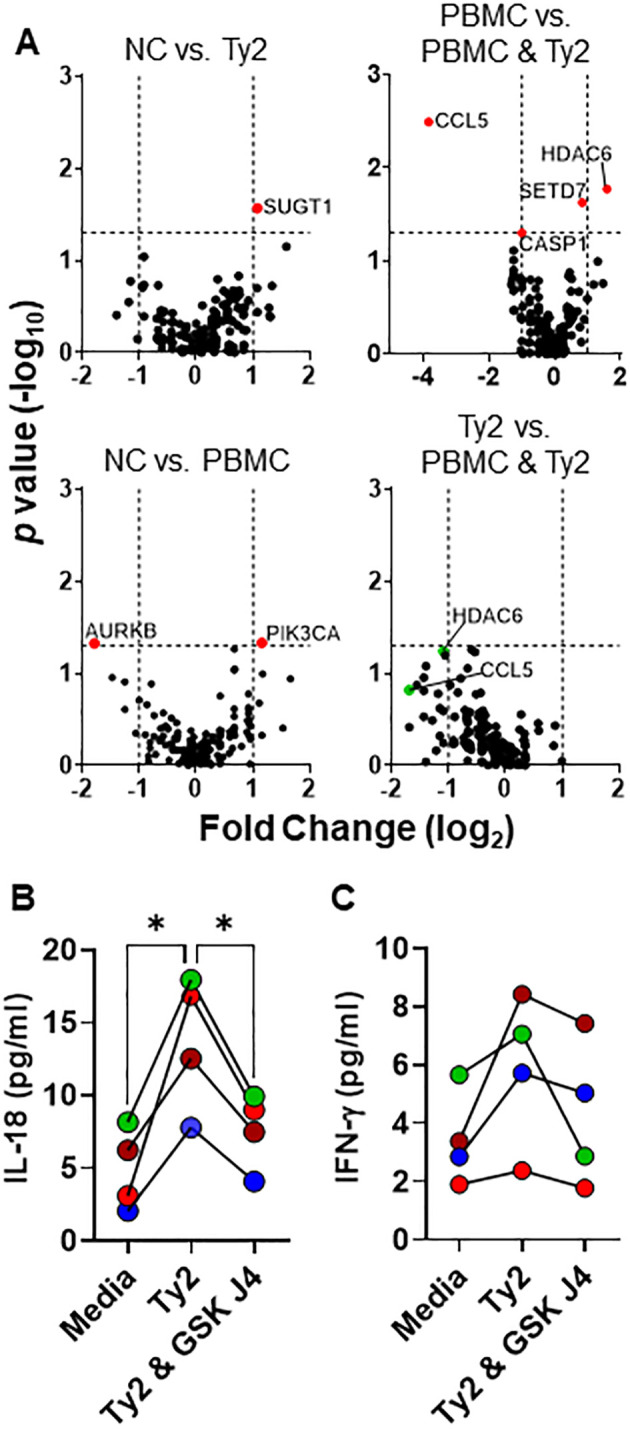
PBMC effects on epithelial cell responses to *S*. Typhi. HODIM were left untreated (NC) or exposed to *S*. Typhi strain Ty2 (Ty2) in the presence or absence of PBMC. After 16 hours, epithelial cells and supernatants were harvested and used to determine **(A)** anti-bacterial and epigenome profile by qRT-PCR. In some experiments, EC & PBMC co-cultures exposed to *S*. Typhi were treated with H3K27 demethylase inhibitor GSK-J4 (10μM) and the supernatants used to measure **(B)** IL-18 (upper chamber) and **(C)** IFN-γ (lower chamber) secretion. Dots are color coded per experiment. Data are representative of 4 independent experiments. *, *P* values < 0.05 were considered statistically significant.

To further assess EC-immune cell crosstalk, we examined the impact of immune cells on the gene regulation of EC in response to *S*. Typhi exposure using the HODIM as described above. After 16 hours, we harvested ECs from the upper chamber and performed gene profiling of chromatin-modifying enzymes and anti-bacterial responses using qRT-PCR. Among the 168 genes studied, only histone deacetylase-6 (HDAC6) and Chemokine (C-C motif) ligand 5 (CCL5) exhibited significant and >2-fold changes in PBMC & EC co-cultures treated with *S.* Typhi compared to untreated co-cultures (i.e., PBMC vs. PBMC & Ty2), indicating their impact on the crosstalk between PBMC and EC in response to *S*. Typhi (**Figure 2A**). Interestingly, while HDAC6 gene expression increases, CCL5 expression decreases in these cultures. No other statistically significant and > 2-fold changes were observed among the other groups. Given the role of HDAC6 in the clearance of infected cells and secretion of IFN-γ (47–49), in some experiments, EC & PBMC co-cultures exposed to *S*. Typhi were treated with the H3K4me3/H3K27me3 demethylase inhibitor GSK-J4 (10μM) and the supernatant used to measure IL-18 (upper chamber) and IFN-γ (lower chamber) secretion using the LegendPlex assay. We found that GSK-J4 significantly reduced the production of IL-18 but only minimal effects, if any, on IFN-γ in co-cultures exposed to *S*. Typhi strain Ty2 (Ty2) (Ty2 vs. Ty2 & GSK J4, *p* = 0.1335) (**Figures 2B, C**.). Thus, IL-18 production might be significantly impacted by H3K4me3 and H3K27me3 chromatin changes.

### Epithelial cell impact on INL epigenome

To gain additional insights into EC-immune cell crosstalk mechanisms, we evaluated the effects of EC on the chromatin changes of the MAIT, NK, and TCR-γδ cells after exposure to *S*. Typhi. To this end, HODIM were left untreated or exposed to *S*. Typhi in the presence or absence of EC. We focus on epigenetic marks, which mediate transcription of IFN gene (*i.e.*, H3K27me3 and H3K4me3) (29, 30). Culture conditions followed the ones described in **Figure 1A** (top and middle panels). After 16 hours, PBMC harvested from the lower chamber of the HODIM model were used to perform immune staining to detect NK (CD45+CD3-CD19-CD56+), MAIT (CD45+CD3+CD19-TCR-γδ-CD161+TCR Vα7.2+), and TCR-γδ (CD45+CD3+CD19-TCR-γδ+) (**Supplementary Figure 2**), and their expression of IFN-γ and TNF-α as well as H3K27me3 and H3K4me3 chromatin changes determined by EpiTOF assays (**Supplementary Table 1**). EpiTOF is a mass cytometry-based analytical platform simultaneously focusing on epigenetic and immunological markers at the single-cell level (15, 39, 40). EpiTOF can interrogate millions of individual cells for epigenetic changes concurrently and has been validated in our lab (15).

**Figure 3:**
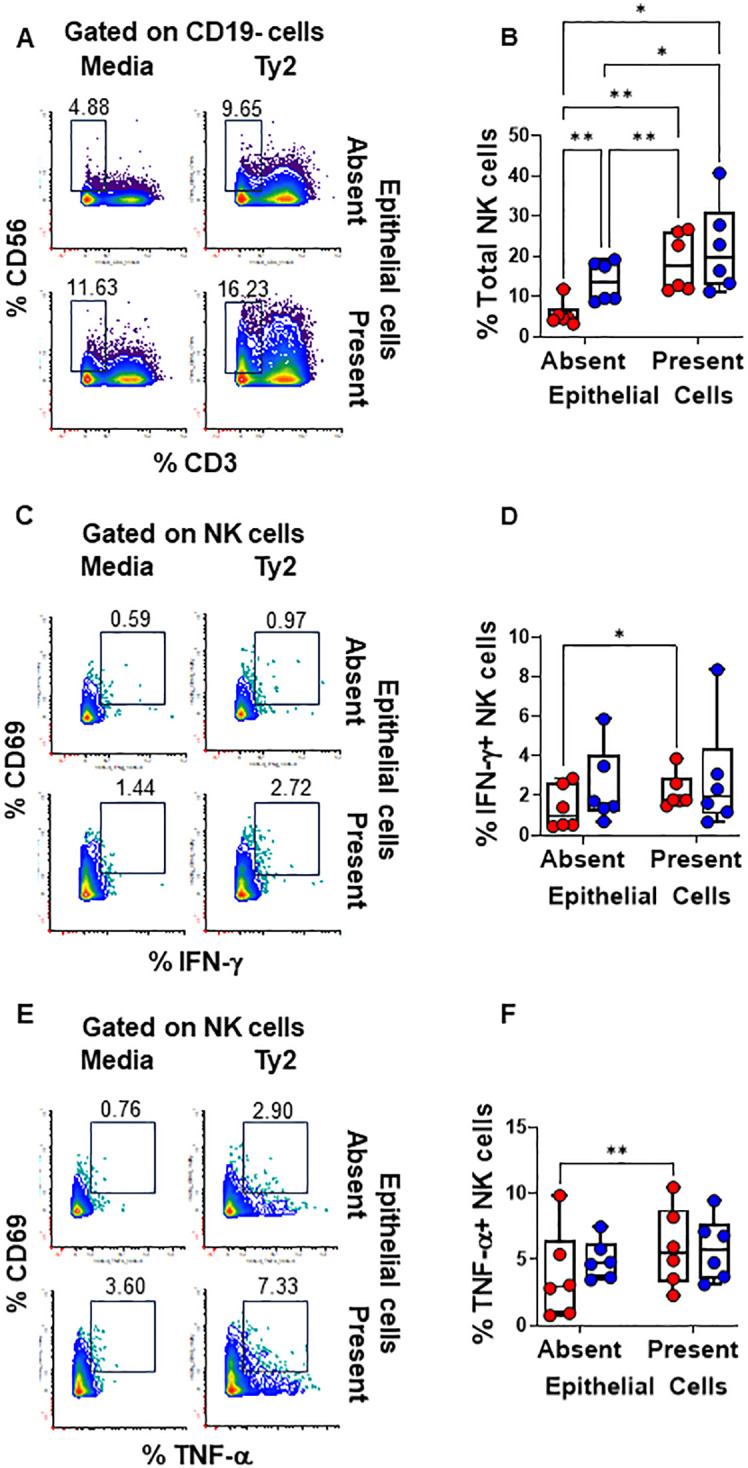
Epithelial cell effects on NK cell responses to *S*. Typhi. HODIM were left untreated (⬤, media only) or exposed to *S*. Typhi strain Ty2 (⬤) in the presence or absence of epithelial cells. After 16 hours, PBMC from the lower chamber were harvested to perform EpiTOF analyses **(A-F)**. Levels of total NK cells **(A, B)**, or their subsets: IFN-γ+ **(C, D)** and TNF-α+ NK cells **(E, F)**. Mixed-effects models were used to compare groups. Data are representative of 6 independent experiments. *P* values < 0.05 were considered statistically significant. *P* values < 0.05 were considered statistically significant. The levels of significance are: *, 0.01 to 0.05; **, 0.001 to 0.01; ***, 0.0001 to 0.001; ****<0.0001.

**Figure 4:**
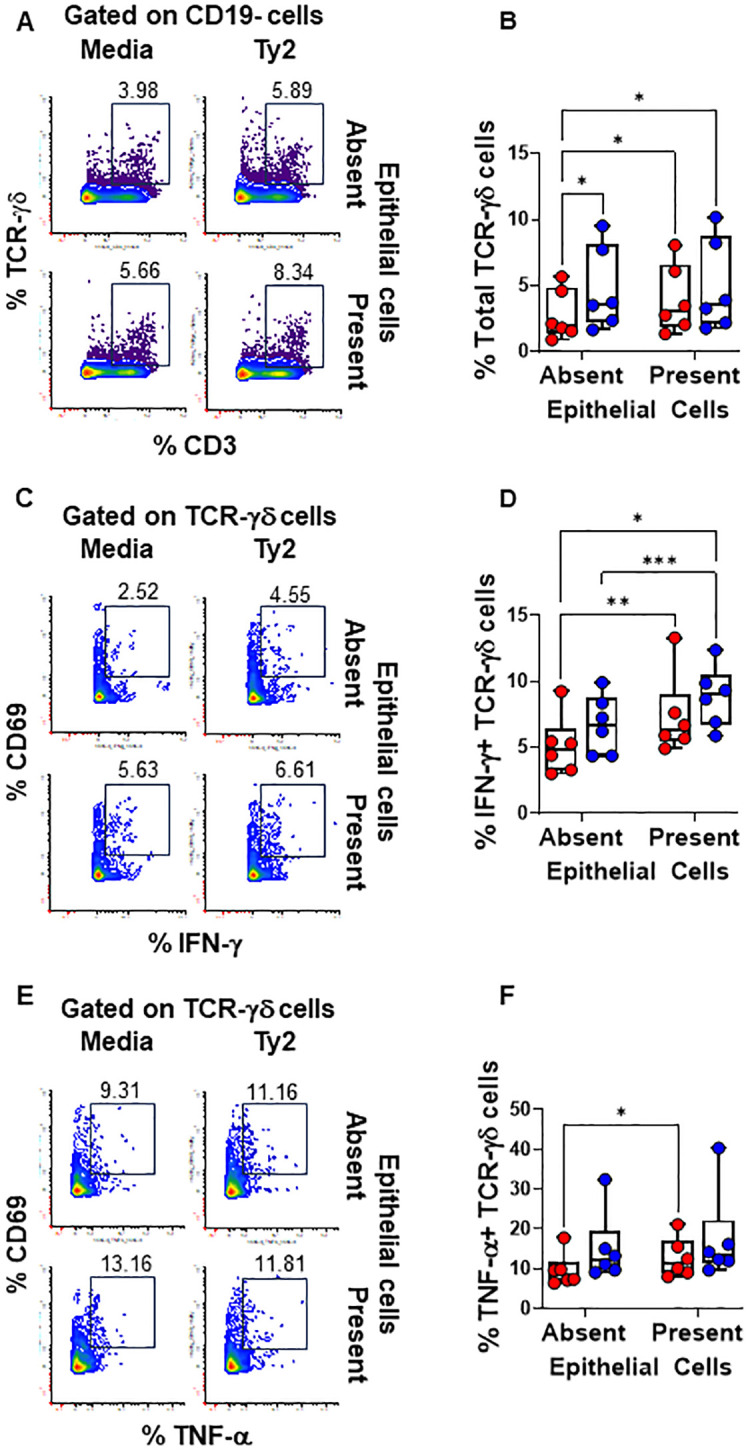
Epithelial cell effects on TCR-γδ cell responses to *S*. Typhi. HODIM were left untreated (⬤, media only) or exposed to *S*. Typhi strain Ty2 (⬤) in the presence or absence of epithelial cells. After 16 hours, PBMC from the lower chamber were harvested to perform EpiTOF analyses **(A-F)**. Levels of total NK cells **(A, B)**, or their subsets: IFN-γ+ **(C, D)** and TNF-α+ NK cells **(E, F)**. Mixed-effects models were used to compare groups. Data are representative of 6 independent experiments. *P* values < 0.05 were considered statistically significant. *P* values < 0.05 were considered statistically significant. The levels of significance are: *, 0.01 to 0.05; **, 0.001 to 0.01; ***, 0.0001 to 0.001; ****<0.0001.

**Figure 5:**
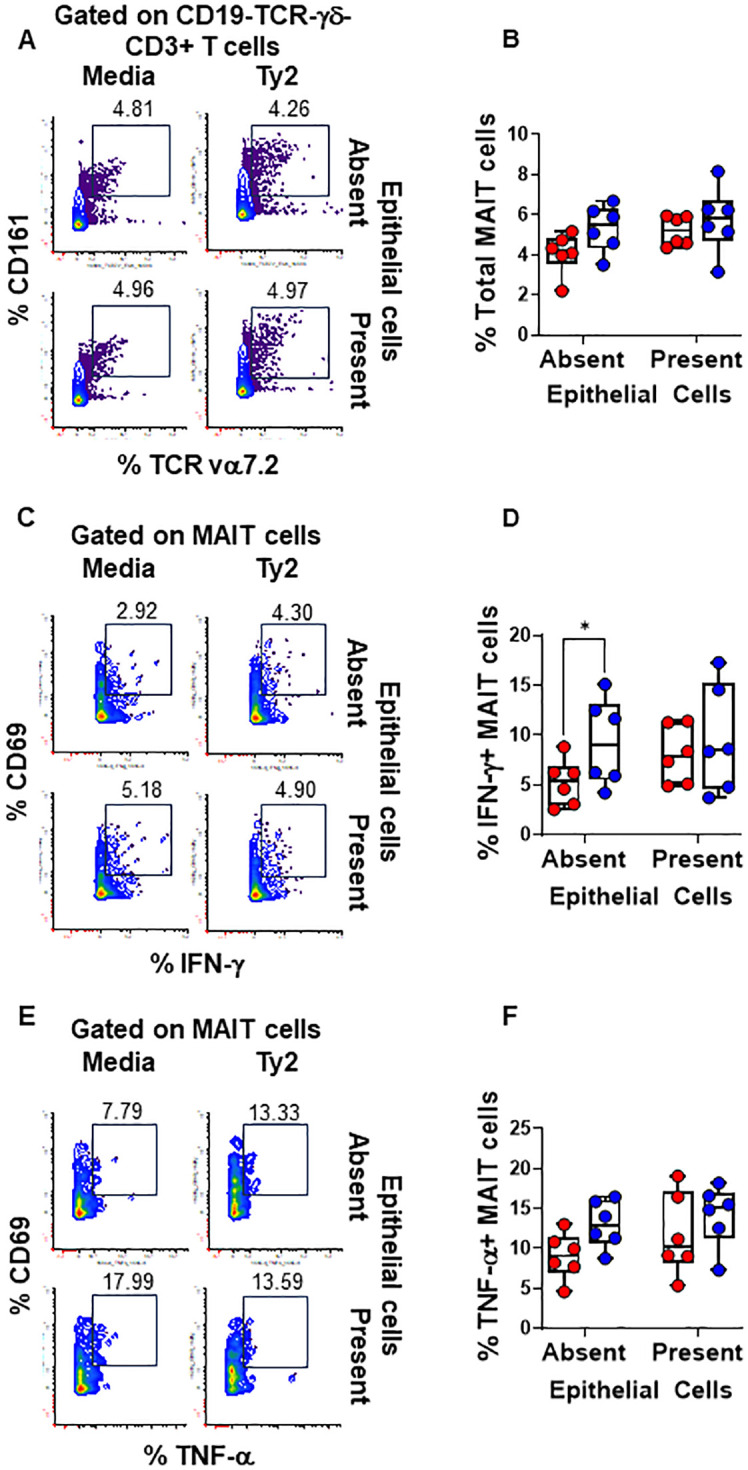
Epithelial cell effects on MAIT cell responses to *S*. Typhi. HODIM were left untreated (⬤, media only) or exposed to *S*. Typhi strain Ty2 (⬤) in the presence or absence of epithelial cells. After 16 hours, PBMC from the lower chamber were harvested to perform EpiTOF analyses **(A-F)**. Levels of total NK cells **(A, B)**, or their subsets: IFN-γ+ **(C, D)** and TNF-α+ NK cells **(E, F)**. Mixed-effects models were used to compare groups. Data are representative of 6 independent experiments. *P* values < 0.05 were considered statistically significant. *P* values < 0.05 were considered statistically significant. The levels of significance are: *, 0.01 to 0.05; **, 0.001 to 0.01; ***, 0.0001 to 0.001; ****<0.0001.

We found that PBMC cultures exposed to *S*. Typhi in the absence of EC showed increased levels of NK (**Figures 3A, B**) and TCR-γδ (**Figures 4A, B**) cells compared to cultures with media only. However, their levels were consistently lower when EC were present (**Figures 3A, B**, **4A, B**). No such changes were observed in MAIT cells (**Figures 5A, B**). In addition, we observed that in the absence of EC, IFN-γ-expressing MAIT cells were higher in cultures exposed to *S*. Typhi compared to control cultures with media only (**Figures 5C, D**). Interestingly, NK and TCR-γδ cells expression of IFN-γ and TNF-α were significantly higher in control cultures (media only) in the presence of EC compared to those cultures in the absence of EC. These results collectively suggest a cooperative role of EC in up-regulating the expression of pro-inflammatory cytokines such as IFN-γ and TNF-α in NK and TCR-γδ cells, regardless of the infection (**Figures 3C-F** and **Figures 4C–F**). As for the frequency of the cells, no such up-regulation of IFN-γ and TNF-α expression was observed in MAIT cells (**Figures 5C–F**).

**Figure 6:**
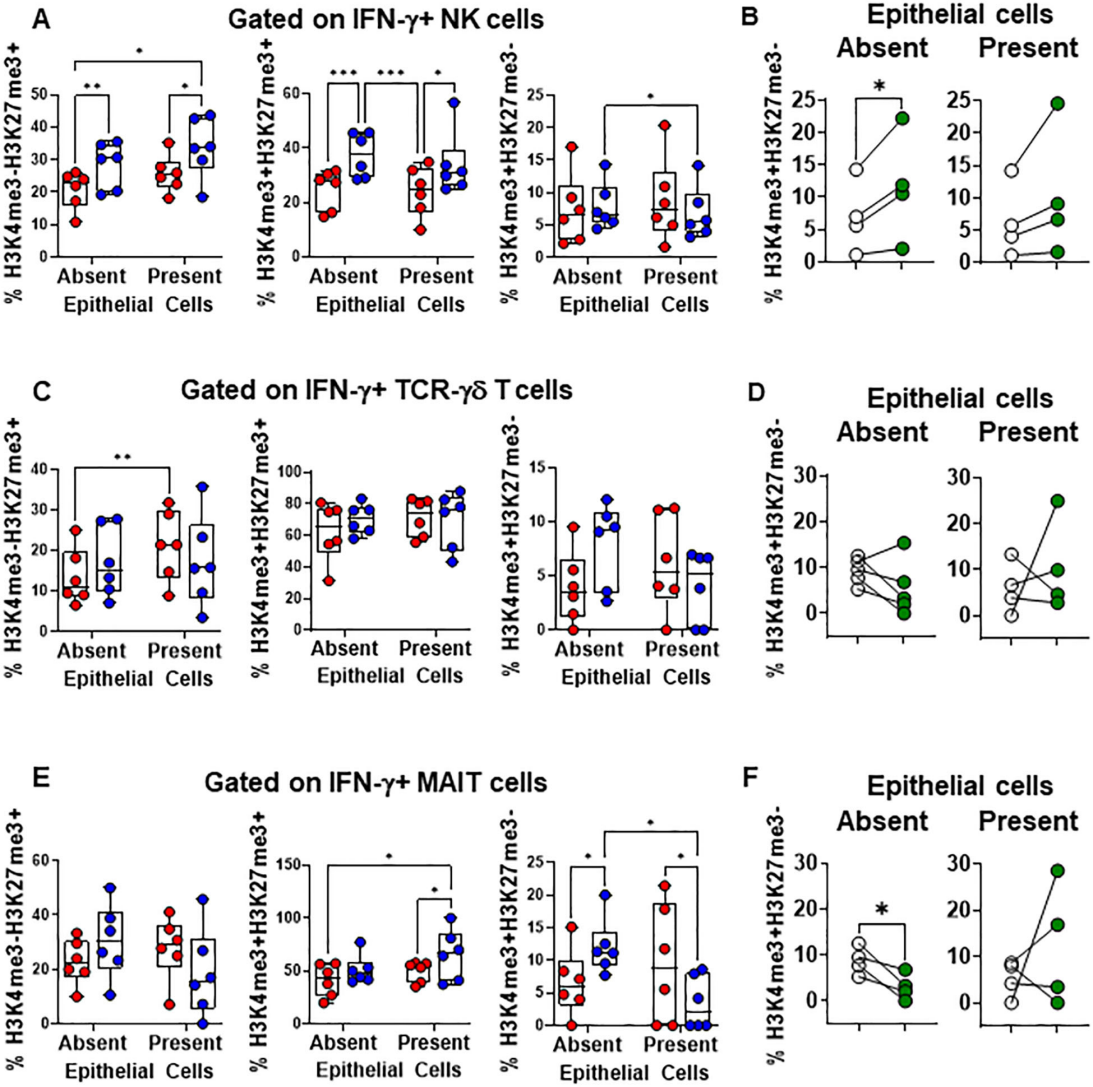
Epithelial cell effects on H3K4me3 and H3K27me3 marks in INLs exposed to *S*. Typhi. HODIM were left untreated (⬤, media only) or exposed to *S*. Typhi strain Ty2 (⬤) in the presence or absence of EC. After 16 hours, PBMC from the lower chamber were harvested to perform EpiTOF analyses **(A-F)**. Levels of H3K4me3 and H3K27me3 marks in IFN-γ+ INLs (NK cells **(A)**, TCR-γδ cells **(C)**, and MAIT cells **(E)**. Data are representative of 6 independent experiments. In some experiments, EC-PBMC co-cultures exposed to *S*. Typhi were treated with H3K27 demethylase inhibitor GSK-J4 (10μM) (⬤) or not (0) and concomitant changes in H3K4me3 and H3K27me3 marks in IFN-γ+ INLs (NK cells **(B)**, TCR-γδ cells **(D)**, and MAIT cells **(F)** were analyzed. Mixed-effects models were used to compare groups. Data are representative of 4 independent experiments. *P* values < 0.05 were considered statistically significant. *P* values < 0.05 were considered statistically significant. The levels of significance are: *, 0.01 to 0.05; **, 0.001 to 0.01; ***, 0.0001 to 0.001; ****<0.0001.

Subsequently, we examined the levels of H3K4me3 and H3K27me3 epigenetic marks in NK, TCR-γδ, and MAIT cells expressing IFN-γ. We found that in PBMC cultures exposed to *S*. Typhi in the absence of EC, there were increased levels of NK (**Figure 6A**) and MAIT cells (**Figure 6E**), and to a lesser extent, TCR-γδ cells (*p* = 0.0897) (**Figure 6C**) expressing H3K4me3 but not H3K27me3 marks compared to cultures in the presence of EC. Interestingly, while the levels of H3K4me3+H3K27me3- NK cells significantly increased in cultures in the absence of EC with *S*. Typhi after GSK-J4 treatment, (**Figure 6B**), the opposite effect was observed for TCR-γδ (**Figure 6D**, *p* = 0.1787) and MAIT cells (**Figure 6F**). GSK-J4 had no or minimal effects in PBMC cultures in the presence of EC (NK, *p* = 0.1428; TCR-γδ, *p* = 0.5674; MAIT cells, *p* = 0.4381) (**Figure 6F**). To further validate these responses, we evaluated the expression levels of H3K4me3 and H3K27me3 in resident MAIT, NK, and TCR-γδ cells isolated from the terminal ileum after exposure to *S*. Typhi. We found that, although there were differences in the expression of H3K4me3 and H3K27me3 marks between migrating and resident INLs, similar changes were observed in INLs expressing H3K4me3 but not H3K27me3 (**Supplementary Figure 3**). Using the same H3K4me3 and H3K27me3 antibodies (same vendor and catalogue number), we also confirmed the antibodies’ specificity by western blot (**Supplementary Figure 4**). Finally, since previous studies have shown an association between IL-15 and IFN- γ production by INLs, such as NK and TCR-γδ cells (50–53), we measured the levels of IL-15 in supernatants from the HODIM lower chambers. We found no significant differences among the groups and no correlations between levels of IL-15 and IFN-γ (**Supplementary Figure 5**). These results suggest that NK cells might be in a different stage of immune response compared to TCR-γδ and MAIT cells at the evaluated time-point. Additionally, these findings suggest that the impact of EC on IFN-γ production in the intestine following *S*. Typhi infection is likely limited due to the low frequency of cells expressing H3K4me3 but not H3K27me3 marks.

## Discussion

This study addressed three important questions. First, how epigenetic changes are related to the production of IFN-γ by NK, TCR-γδ, and MAIT cells in a human gut microenvironment. Second, most studies reflect long-term stimulation of the cells; however, to our knowledge, there are no reports showing how different histone modifications control the production of cytokines soon after exposure to pathogenic bacteria such as *S*. Typhi in human cells. Third, how the crosstalk between epithelial cells and NK, TCR-γδ, and MAIT cells affects IFN-γ production and the epigenetic marks such as H3K4me3 and H3K27me3. This study builds on our previous findings that MAIT subsets exhibiting distinct cytokine patterns (*i.e.*, IFN-γ<συπ>+</sup> TNF-α<συπ>+</sup> IL-17A^-^ positive cells) were associated with protection against typhoid fever (25), and that *S*. Typhi infection regulates changes in chromatin marks that depend on individual cell subsets.

Intestinal epithelial cells can indirectly interact with innate immune cells by releasing cytokines, chemokines, hormones, and enzymes (9, 54, 55). Here, we found that EC are critical in downregulating IFN-γ, TNF-α, IL-10, and IL-23 secretion by PBMC, independently of *S*. Typhi infection. This underscores the significance of EC in maintaining homeostasis and preventing hyperstimulation of immune cells in the intestine in non-infectious settings. Since previous studies have indicated that GSK-J4 can reduce inflammatory responses in the gut (56, 57), including IFN-γ secretion by NK cells (58), we sought to confirm the GSK-J4 anti-inflammatory properties in our experimental settings of *S*. Typhi infection. Surprisingly, we found that the GSK-J4 treatment significantly reduced the production of IL-18 but had only minimal effects IFN-γ in co-cultures exposed to *S*. Typhi. We hypothesize that these unexpected effects might be context-dependent, likely influenced by the microenvironment, cell type, and stage of immune response. For example, IL-18 inhibition may lead to compensatory mechanisms or alternative pathways for IFN-γ production that are not affected by the inhibition of IL-18 signaling (59). Concomitantly and/or alternatively, the timing and duration of cytokine inhibition may also play a role (59). Short-term inhibition may not fully reflect the long-term effects on cytokine production, and compensatory mechanisms may take additional time to manifest (59). Of note, our results agree with previous studies showing that in a mouse model of inflammatory colitis, GSK-J4 reduced gut inflammation without affecting IFN-γ production in gut mucosa (57).

We also found that among the three cell subsets, NK, TCR-γδ, and MAIT cells, only MAIT cells showed, in the absence of EC, significant increases in IFN-γ expression in cultures exposed to *S*. Typhi compared to control cultures with media only. However, when comparing the levels of H3K4me3 and H3K27me3 marks in NK, TCR-γδ, and MAIT cells expressing IFN-γ, we found that NK and MAIT cells, and to a lesser extent TCR-γδ cells, had decreased levels of the H3K4me3 but not H3K27me3 mark in co-cultures of PBMC & ECs compared to PBMC-only cultures following *S*. Typhi infection, suggesting that EC might play a role in the changes of H3K4me3 marks in response to *S.* Typhi in the human gut. Although the impact of EC on IFN-γ production in the intestine following S. Typhi infection is likely limited due to the low frequency of cells expressing H3K4me3 but not H3K27me3 marks, based on results of treatment with GSK-J4, we cannot rule out that EC might indirectly impact chromatin changes, and consequently IFN-γ expression, by NK, TCR-γδ, and MAIT cells through the regulation of secretion of IL-18. GSK-J4 can reverse histone methylation by opposing histone lysine demethylase (KDM), which removes methylation marks such as H3K4me3 and H3K27me3 (60). Depending on which lysine residue is targeted, a given KDM can either activate or repress gene transcription (61). For example, actively transcribed regions of DNA are associated with H3K4me3 methylation marks and drive gene expression, whereas H3K27me3 is a mark of heterochromatin and suppresses gene expression (62, 63). Thus, it is reasonable to hypothesize that H3K4me3 and H3K27me3 chromatin changes impact IL-18 production. One possibility would be through HDAC6, given its role in the clearance of infected cells and secretion of IFN-γ (47–49). Previous studies support this hypothesis by showing that HDAC inhibitors regulate the expression of IL-18 (64). Interestingly, plasma from animals carrying melanoma previously exposed to HDAC inhibitors displayed elevated levels of CCL5 (65). These studies support our findings showing that while HDAC6 increases, CCL5 decreases in co-cultures of PBMC & EC compared to cultures with PBMC only after *S*. Typhi infection.

Notably, while the level of H3K4me3+ H3K27me3- NK cells significantly increased in cultures without EC with *S*. Typhi after GSK-J4 treatment, the opposite effect was observed for TCR-γδ and MAIT cells. GSK-J4 did not affect PBMC cultures in the presence of ECs. These results suggest that NK cells exhibit differences in the regulation of the H3K4me3 epigenetic mark compared to TCR-γδ and MAIT cells, either because of inherent differences among these cell types, or because they are at a different stage of the immune response at the evaluated time point. Additionally, these findings suggest that NK, TCR-γδ, and MAIT cell production of IFN-γ in the intestine might be modulated through changes in the expression of the H3K4me3 mark following *S.* Typhi infection, with limited or no impact of EC. It is worth noting that TCR-γδ and MAIT cells compete for niches within tissues. Recent evidence suggests that these cells may play a fundamental role in tissue physiology by acting as a network with overlapping and potentially synergistic functions, rather than functioning solely as individual subsets (9).

In summary, we demonstrated that NK and MAIT cells, and to a lesser extent TCR-γδ cells, expressing IFN-γ changed the levels of their H3K4me3 chromatin marks following *S.* Typhi infection, suggesting that these marks play a role in the human gut host immune response to *S.* Typhi. We also showed that although EC had little or no impact on the H3K4me3 changes, they might indirectly influence chromatin changes by regulating epithelial cell secretion of IL-18 through the HDAC6 gene. Further experiments using resident immune cells from the intestine are needed to confirm and extend these observations.

## Data Availability

The original contributions presented in the study are included in the article/**Supplementary Material**, further inquiries can be directed to the corresponding author/s.
