# Supplementary material for: Epigenetic regulation in epithelial cells and innate lymphocyte responses to S. Typhi infection: insights into IFN-γ production and intestinal immunity
Source: Front Immunol. 2024 Sep 20;15:1448717. doi: 10.3389/fimmu.2024.1448717 (PMC11450450; doi:10.3389/fimmu.2024.1448717)
Supplement: Supplementary file 1 [file DataSheet1.pdf]

Intestinal epithelial cells drive epigenetic changes of innate T cells during Salmonella enterica serovar Typhi infection

Rosângela Salerno-Goncalves, Haiyan Chen, Andrea C. Bafford, and Marcelo B. Sztein

Supplementals

Supplemental Table 1. Anitbodies used in this manuscript studies

	Atomic Mass:	Marks/Markers	Vendor	Catalog #
1	89 Y	CD45	Standard BioTools	3089003B
2	113 Cd	CD19	Biolegend	302247
3	114 Cd	CD3	Biolegend	300443
5	142 Nd	β2 CD298	Biolegend	316302/ 341712
6	143 Nd	H3K4me3	Biolegend	608804
7	145 Nd	β2 CD298	Biolegend	316302/ 341712
8	152 Sm	TCR$\gamma\delta$	Standard BioTools	3152008B
9	154 Sm	TNF-α	Biolegend	502941
10	158 Gd	IFN-γ	Standard BioTools	3158017B
11	159 Tb	CD161	Standard BioTools	3159004B
12	162Dy	CD69	Standard BioTools	3162001B
13	163 Dy	H3K27me3	ActiveMotif	91167
14	164 Dy	β2 CD298	Biolegend	316302/ 341712
15	165 Ho	TCR Vα 7.2	Biolegend	351702
16	166 Er	β2 CD298	Biolegend	316302/ 341712
17	176 Yb	CD56	Standard BioTools	3176001B
18	195 Pt	H3	Biolegend	687602
	**Atomic Mass:**	**Other:**		
19	198 Pt	Live/dead	Standard BioTools	201198
20	N/A	"Cell lengh"		
21	103 Rh	DNA content	Standard BioTools	2011 03B

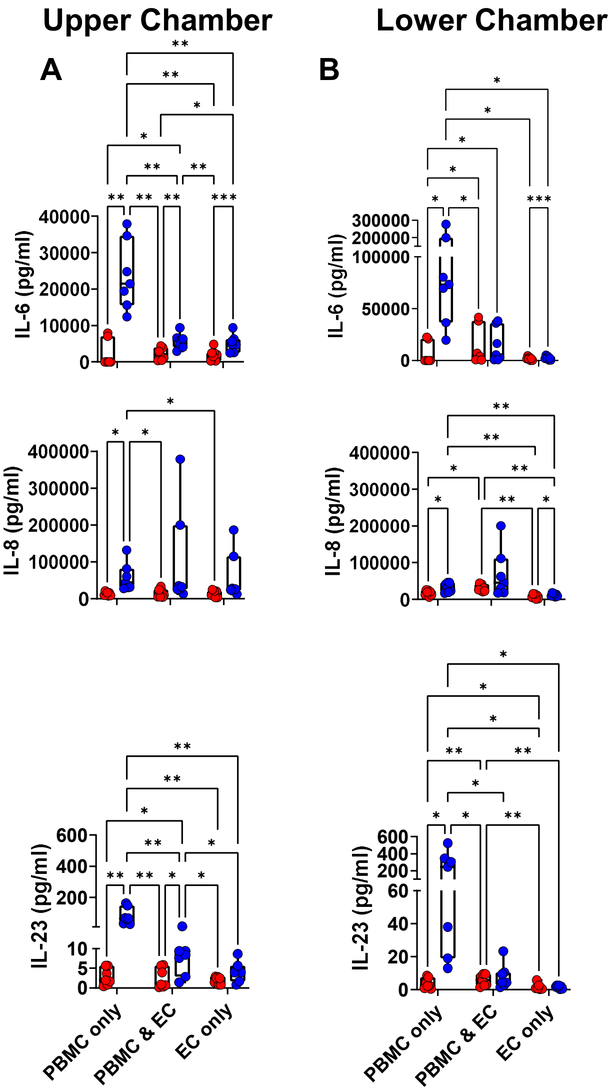

Supplemental Fig. 1. Crosstalk between migrating immune cells and intestinal epithelial cells and the consequences for host responses to *S*. Typhi. (**A-B**) Cultures were left untreated (●, media only) or exposed to *S*. Typhi strain Ty2 (●) in the presence (PBMC only, or PBMC & EC [epithelial cells]) or absence of PBMC (EC only). After 16 hours, supernatants were harvested and used to determine cytokine/chemokine secretion in the upper (**A**) and lower chamber (**B**). Mixed-effects models were used to compare groups. Data are representative of 7 independent experiments. *****, *P* values < 0.05 were considered statistically significant. *P* values < 0.05 were considered statistically significant. The levels of significance are: *****, 0.01 to 0.05; **, 0.001 to 0.01; ***, 0.0001 to 0.001.

Supplemental Fig. 2. Gating strategy to identify cell lineages

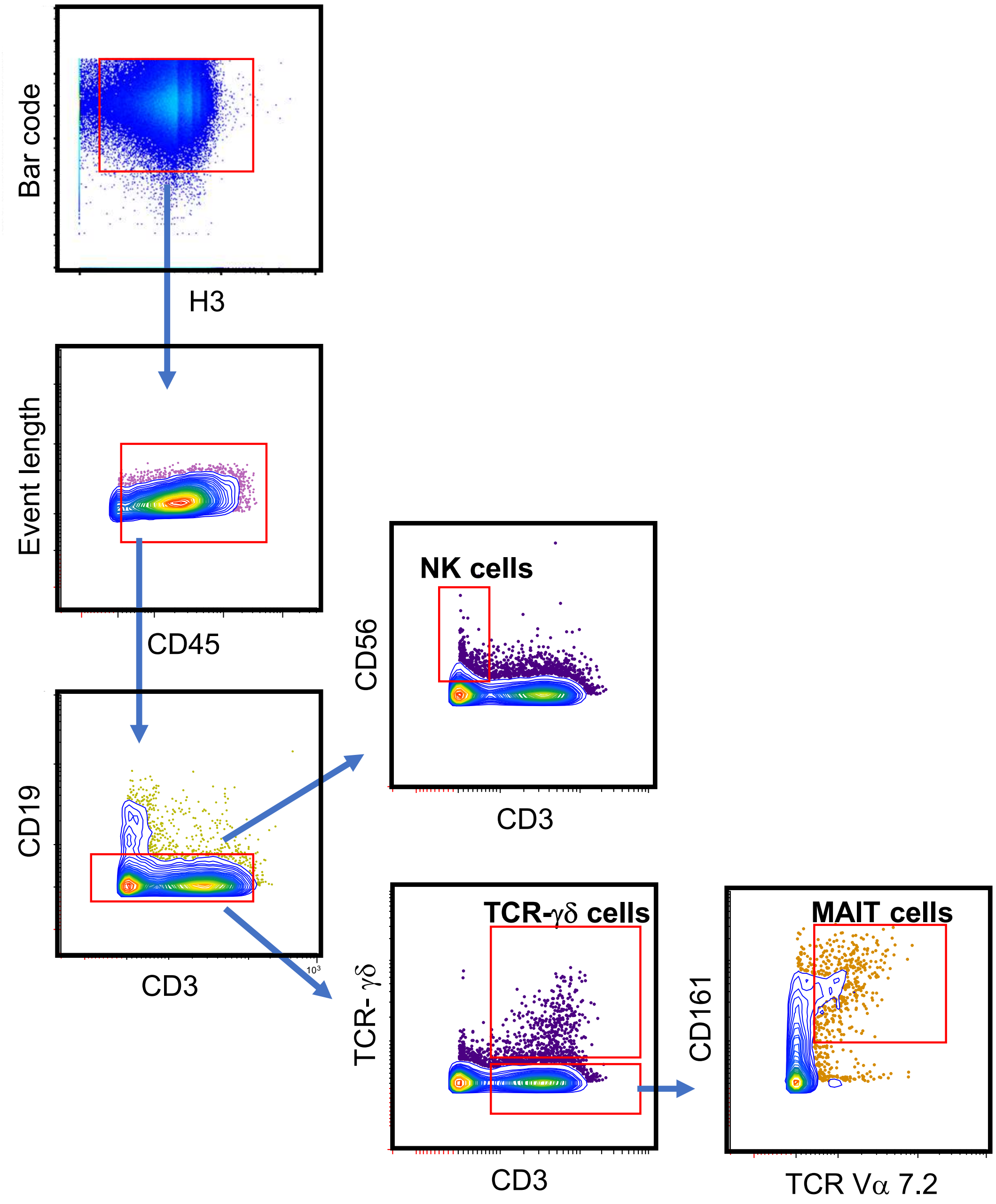

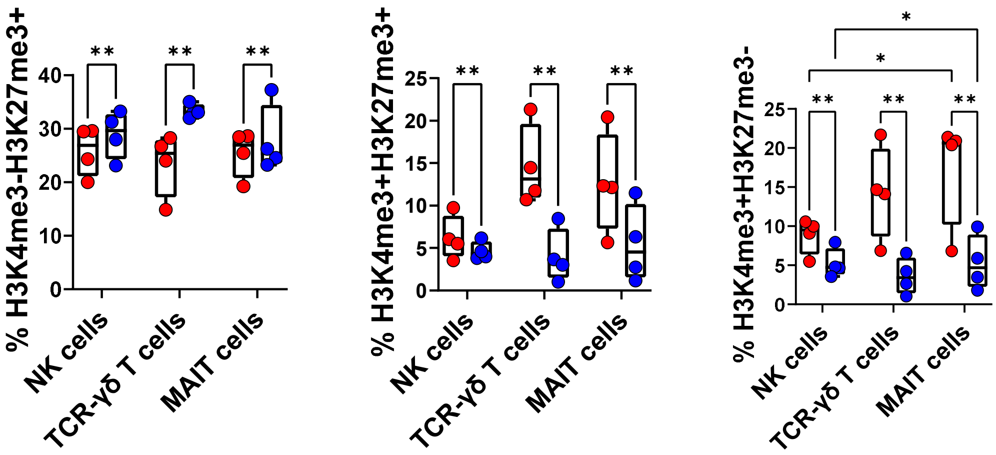

Supplemental Fig. 3. Changes on H3K4me3 and H3K27me3 marks in intestinal INLs exposed to *S.* Typhi. Cells isolated from healthy terminal ileum explants were left untreated (●, media only) or exposed to *S*. Typhi strain Ty2 (●). After 16 hours, cells were harvested to perform EpiTOF analyses. Levels of H3K4me3 and H3K27me3 marks in INLs (NK cells, TCR-γδ cells, and MAIT cells) were evaluated. Data are representative of 4 independent experiments. Mixed-effects models were used to compare groups. *P* values < 0.05 were considered statistically significant. *P* values < 0.05 were considered statistically significant. The levels of significance are: *, *p*= 0.01 to 0.05; **, *p* = 0.001 to 0.01.

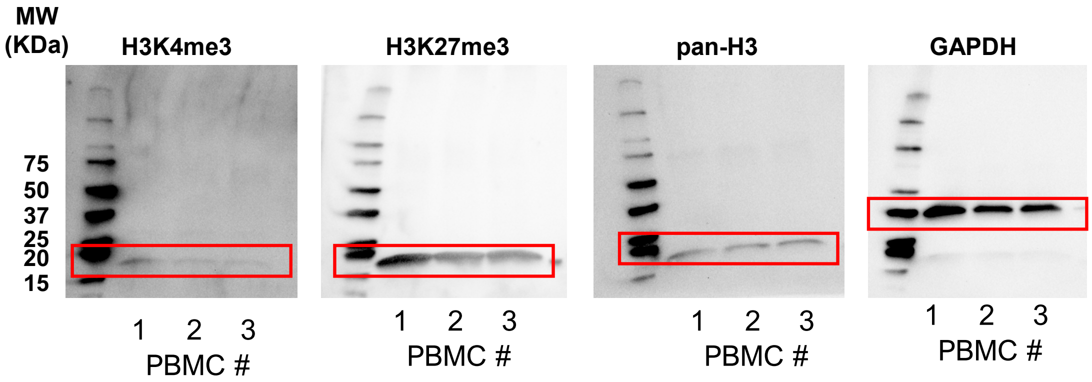

Supplemental Fig. 4. Validation of EpiTOF antibodies on PBMC by western blot. HODIM were left untreated (media only) or exposed to *S*. Typhi strain Ty2 (Ty2) in the presence of EC. After 16 hours, PBMC from 3 different experiments (1-3) were harvested from the lower chamber, lysed, and the expression of H3K4me3 (MW, 17kDa), H3K27me3 (MW, 17kDa), and pan-H3 (MW, 17kDa) were detected by western blot using the same primary antibodies as the one used for EpiTOF. GAPDH (MW, 39kDa) antibodies were used as controls. MW, molecular weight.

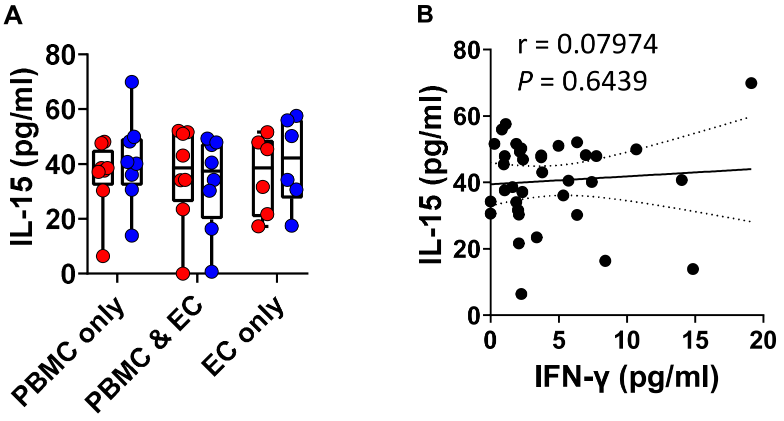

Supplemental Fig. 5. Crosstalk between migrating immune cells and intestinal epithelial cells and the consequences for host responses to *S.* Typhi. (**A**) Cultures were left untreated (●, media only) or exposed to *S.* Typhi strain Ty2 (●) in the presence (PBMC only, or PBMC & EC [epithelial cells]) or absence of PBMC (EC only). After 16 hours, supernatants from the lower chambers were harvested and used to determine IL-15 secretion. Mixed-effects models were used to compare groups, and no significant differences were found among the groups. (**B**) Correlation between levels of IL-15 and IFN-γ. Data are representative of up to 8 replicates from 4 independent experiments. The solid line represents the trendline. Dashed lines represent 95% confidence intervals. Shown are the coefficient of determination "r" and the "*P*" value. Correlations used the two-sided Pearson Product Moment tests.
