# Supplementary material for: Epigenetic regulation in epithelial cells and innate lymphocyte responses to S. Typhi infection: insights into IFN-γ production and intestinal immunity
Source: Front Immunol. 2024 Sep 20;15:1448717. doi: 10.3389/fimmu.2024.1448717 (PMC11450450; doi:10.3389/fimmu.2024.1448717)
Supplement: Supplementary file 2 [file DataSheet2.pdf]

Supplemental Table 2

PCR Array Cat. #: PAHS-085Z RT^2 Profiler™ PCR Array Human Epigenetic Chromatin Modification Enzymes

Position	Unigene	Refseq	Symbol	Description	Gname	RT2 Catalog
A01	Hs.491060	NM_018489	ASH1L	Ash1 (absent, small, or homeotic)-like (Drosophila)	ASH1/ASH1L1/KMT2H	PPH21292B
A02	Hs.592510	NM_001880	ATF2	Activating transcription factor 2	CRE-BP1/CREB-2/CREB2/HB16/TREB7	PPH00071A
A03	Hs.250822	NM_003600	AURKA	Aurora kinase A	AIK/ARK1/AURA/AURORA2/BTAK/PPP1R47/STK15/STK6	PPH15095A
A04	Hs.442658	NM_004217	AURKB	Aurora kinase B	AIK2/AIM-1/AIM1/ARK2/AurB/IPL1/PPP1R48/STK12/STK5	PPH21059F
A05	Hs.98338	NM_003160	AURKC	Aurora kinase C	AIE2/AIK3/ARK3/AurC/HEL-S-90/SPGF5/STK13/aurora-C	PPH10708A
A06	Hs.720136	NM_199141	CARM1	Coactivator-associated arginine methyltransferase 1	PRMT4	PPH05900A
A07	Hs.269092	NM_004824	CDYL	Chromodomain protein, Y-like	CDYL1	PPH08073A
A08	Hs.701991	NM_000246	CIITA	Class II, major histocompatibility complex, transactivator	C2TA/CIITAIV/MHC2TA/NLRA	PPH00419A
A09	Hs.488051	NM_020536	KAT14	Lysine acetyltransferase 14	ATAC2/CRP2BP/CSRP2BP/PRO1194/dJ717M23.1	PPH14458A
A10	Hs.202672	NM_001379	DNMT1	DNA (cytosine-5-)-methyltransferase 1	ADCADN/AIM/CXXC9/DNMT/HSN1E/MCMT	PPH01055F
A11	Hs.515840	NM_022552	DNMT3A	DNA (cytosine-5-)-methyltransferase 3 alpha	DNMT3A2/M.HsaIIIA/TBRS	PPH02339B
A12	Hs.643024	NM_006892	DNMT3B	DNA (cytosine-5-)-methyltransferase 3 beta	ICF/ICF1/M.HsaIIIB	PPH01054F
B01	Hs.713641	NM_032482	DOT1L	DOT1-like, histone H3 methyltransferase (S. cerevisiae)	DOT1/KMT4	PPH16214A
B02	Hs.409210	NM_014648	DZIP3	DAZ interacting protein 3, zinc finger	PPP1R66/UURF2/hRUL138	PPH19450A
B03	Hs.709218	NM_006709	EHMT2	Euchromatic histone-lysine N-methyltransferase 2	BAT8/C6orf30/G9A/GAT8/KMT1C/NG36	PPH09942A
B04	Hs.464733	NM_052911	ESCO1	Establishment of cohesion 1 homolog 1 (S. cerevisiae)	A930014I12Rik/CTF/ECO1/EFO1/ESO1	PPH16227B
B05	Hs.99480	NM_001017420	ESCO2	Establishment of cohesion 1 homolog 2 (S. cerevisiae)	2410004I17Rik/EFO2/RBS	PPH10788A
B06	Hs.632532	NM_003642	HAT1	Histone acetyltransferase 1	KAT1	PPH01563E
B07	Hs.88556	NM_004964	HDAC1	Histone deacetylase 1	GON-10/HD1/RPD3/RPD3L1	PPH01735F
B08	Hs.26593	NM_032019	HDAC10	Histone deacetylase 10	HD10	PPH06896A
B09	Hs.744132	NM_024827	HDAC11	Histone deacetylase 11	HD11	PPH06921A
B10	Hs.3352	NM_001527	HDAC2	Histone deacetylase 2	HD2/RPD3/YAF1	PPH01717F
B11	Hs.519632	NM_003883	HDAC3	Histone deacetylase 3	HD3/RPD3/RPD3-2	PPH05911F
B12	Hs.20516	NM_006037	HDAC4	Histone deacetylase 4	AHO3/BDMR/HA6116/HD4/HDAC-4/HDAC-A/HDACA	PPH05912F
C01	Hs.438782	NM_005474	HDAC5	Histone deacetylase 5	HD5/NY-CO-9	PPH05913C
C02	Hs.6764	NM_006044	HDAC6	Histone deacetylase 6	CPBHM/HD6/JM21/PPP1R90	PPH01475B
C03	Hs.200063	NM_001098416	HDAC7	Histone deacetylase 7	HD7/HD7A/HDAC7A	PPH05914A
C04	Hs.310536	NM_018486	HDAC8	Histone deacetylase 8	CDA07/CDLS5/HD8/HDACL1/MRXS6/RPD3/WTS	PPH06870B
C05	Hs.196054	NM_178425	HDAC9	Histone deacetylase 9	HD7/HD7b/HD9/HDAC/HDAC7/HDAC7B/HDAC9B/HDAC9FL	PPH06942F
C06	Hs.463045	NM_021078	KAT2A	K(lysine) acetyltransferase 2A	GCN5/GCN5L2/PCAF-b/hGCN5	PPH10838F
C07	Hs.533055	NM_003884	KAT2B	K(lysine) acetyltransferase 2B	CAF/P/CAF/PCAF	PPH02176F
C08	Hs.397010	NM_006388	KAT5	K(lysine) acetyltransferase 5	ESA1/HTATIP/HTATIP1/PLIP/TIP/TIP60/ZC2HC5/cPLA2	PPH01029A
C09	Hs.491577	NM_006766	KAT6A	K(lysine) acetyltransferase 6A	MOZ/MRD32/MYST-3/MYST3/RUNXBP2/ZC2HC6A/ZNF220	PPH10514B
C10	Hs.35758	NM_012330	KAT6B	K(lysine) acetyltransferase 6B	GTPTS/MORF/MOZ2/MYST4/ZC2HC6B/qkf/querkopf	PPH01590F
C11	Hs.21907	NM_007067	KAT7	K(lysine) acetyltransferase 7	HBO1/HBOA/MYST2/ZC2HC7	PPH01582A
C12	Hs.533803	NM_032188	KAT8	K(lysine) acetyltransferase 8	MOF/MYST1/ZC2HC8/hMOF	PPH09171A
D01	Hs.591518	NM_015013	KDM1A	Lysine (K)-specific demethylase 1A	AOF2/BHC110/KDM1/LSD1	PPH17389A
D02	Hs.155983	NM_014663	KDM4A	Lysine (K)-specific demethylase 4A	JHDM3A/JMJD2/JMJD2A/TDRD14A	PPH12994F
D03	Hs.709425	NM_015061	KDM4C	Lysine (K)-specific demethylase 4C	GASC1/JHDM3C/JMJD2C/TDRD14C	PPH14430F
D04	Hs.18891	NM_006618	KDM5B	Lysine (K)-specific demethylase 5B	CT31/JARID1B/PLU-1/PLU1/PPP1R98/PUT1/RBBP2H1A	PPH12457F
D05	Hs.631768	NM_004187	KDM5C	Lysine (K)-specific demethylase 5C	DXS1272E/JARID1C/MRX13/MRXJ/MRXSCJ/MRXSJ/SMCX	PPH10903F
D06	Hs.223678	NM_001080424	KDM6B	Lysine (K)-specific demethylase 6B	JMJD3	PPH10922A
D07	Hs.25674	NM_003927	MBD2	Methyl-CpG binding domain protein 2	DMTase/NY-CO-41	PPH08621A
D08	Hs.258855	NM_005933	KMT2A	Myeloid/lymphoid or mixed-lineage leukemia	ALL-1/CXXC7/HRX/HTRX1/MLL/MLL-AF9/MLL/GAS7/MLL1	PPH15272A
D09	Hs.647120	NM_170606	KMT2C	Myeloid/lymphoid or mixed-lineage leukemia 3	HALR/MLL3	PPH16385A
D10	Hs.592262	NM_182931	KMT2E	Myeloid/lymphoid or mixed-lineage leukemia 5	HDCMC04P/MLL5/NKp44L	PPH18528A
D11	Hs.744921	NM_001085487	MYSM1	Myb-like, SWIRM and MPN domains 1	2A-DUB/2ADUB	PPH20752A
D12	Hs.596314	NM_003743	NCOA1	Nuclear receptor coactivator 1	F-SRC-1/KAT13A/RIP160/SRC1/bHLHe42/bHLHe74	PPH01273F
E01	Hs.592142	NM_181659	NCOA3	Nuclear receptor coactivator 3	ACTR/AIB-1/AIB1/CAGH16/CTG26/KAT13B/RAC3/SRC-3/SRC3	PPH05891A
E02	Hs.368971	NM_014071	NCOA6	Nuclear receptor coactivator 6	AIB3/ASC2/NRC/PRIP/RAP250/TRBP	PPH05909A
E03	Hs.197071	NM_014397	NEK6	NIMA (never in mitosis gene a)-related kinase 6	SID6-1512	PPH18826A
E04	Hs.106861	NM_022455	NSD1	Nuclear receptor binding SET domain protein 1	ARA267/KMT3B/SOTOS/SOTOS1/STO	PPH02289B
E05	Hs.435714	NM_002576	PAK1	P21 protein (Cdc42/Rac)-activated kinase 1	PAKalpha	PPH01505F
E06	Hs.20521	NM_001536	PRMT1	Protein arginine methyltransferase 1	ANM1/HCP1/HRMT1L2/IR1B4	PPH00489A
E07	Hs.154163	NM_001535	PRMT2	Protein arginine methyltransferase 2	HRMT1L1	PPH00490A
E08	Hs.152337	NM_005788	PRMT3	Protein arginine methyltransferase 3	HRMT1L3	PPH00483A
E09	Hs.367854	NM_006109	PRMT5	Protein arginine methyltransferase 5	HRMT1L5/IBP72/JBP1/SKB1/SKB1Hs	PPH17965A
E10	Hs.26006	NM_018137	PRMT6	Protein arginine methyltransferase 6	HRMT1L6	PPH08640A
E11	Hs.679580	NM_019023	PRMT7	Protein arginine methyltransferase 7	-	PPH10487A
E12	Hs.504530	NM_019854	PRMT8	Protein arginine methyltransferase 8	HRMT1L3/HRMT1L4	PPH16603A
F01	Hs.591490	NM_007212	RNF2	Ring finger protein 2	BAP-1/BAP1/DING/HIPI3/RING1B/RING2	PPH11659A
F02	Hs.729085	NM_019592	RNF20	Ring finger protein 20	BRE1/BRE1A/hBRE1	PPH13371B
F03	Hs.445387	NM_004586	RPS6KA3	Ribosomal protein S6 kinase, 90kDa, polypeptide 3	CLS/HU-3/ISPK-1/MAPKAPK1B/MRX19/RSK/RSK2/S6K-alpha3	PPH05685F
F04	Hs.510225	NM_004755	RPS6KA5	Ribosomal protein S6 kinase, 90kDa, polypeptide 5	MSK1/MSPK1/RLPK	PPH01791A
F05	Hs.297483	NM_014712	SETD1A	SET domain containing 1A	KMT2F/Set1/Set1A	PPH16370A
F06	Hs.507122	NM_015048	SETD1B	SET domain containing 1B	KMT2G/Set1B	PPH12941A
F07	Hs.517941	NM_014159	SETD2	SET domain containing 2	HBP231/HIF-1/HIP-1/HSPC069/HYPB/KMT3A/SET2/p231HBP	PPH07461A
F08	Hs.510407	NM_199123	SETD3	SET domain containing 3	C14orf154	PPH16900A
F09	Hs.606200	NM_017438	SETD4	SET domain containing 4	C21orf18/C21orf27	PPH21338A
F10	Hs.288164	NM_001080517	SETD5	SET domain containing 5	-	PPH19191A
F11	Hs.731691	NM_024860	SETD6	SET domain containing 6	-	PPH14081B
F12	Hs.480792	NM_030648	SETD7	SET domain containing (lysine methyltransferase) 7	KMT7/SET7/SET7/9/SET9	PPH13196A
G01	Hs.572262	NM_001324504	KMT5A	Lysine methyltransferase 5A	PR-Set7/PR/SET07/SET07/SET8/SETD8	PPH58095A
G02	Hs.643565	NM_012432	SETDB1	SET domain, bifurcated 1	ESET/H3-K9-HMTase4/KG1T/KMT1E/TDRD21	PPH17319C
G03	Hs.631789	NM_031915	SETDB2	SET domain, bifurcated 2	C13orf4/CLLD8/CLLL8/KMT1F	PPH21336A
G04	Hs.567571	NM_022743	SMYD3	SET and MYND domain containing 3	KMT3E/ZMYND1/ZNFN3A1/bA74P14.1	PPH07594B
G05	Hs.522639	NM_003173	SUV39H1	Suppressor of variegation 3-9 homolog 1 (Drosophila)	H3-K9-HMTase 1/KMT1A/MG44/SUV39H	PPH09088A
G06	Hs.632120	NM_001300909	KMT5B	Lysine methyltransferase 5B	CGI-85/CGI85/MRD51/SUV420H1	PPH21055A
G07	Hs.379466	NM_003336	UBE2A	Ubiquitin-conjugating enzyme E2A	HHR6A/MRXS30/MRXSN/RAD6A/UBC2	PPH02713B
G08	Hs.730071	NM_003337	UBE2B	Ubiquitin-conjugating enzyme E2B	E2-17kDa/HHR6B/HR6B/RAD6B/UBC2	PPH02743A
G09	Hs.99819	NM_006447	USP16	Ubiquitin specific peptidase 16	UBP-M/UBPM	PPH10799A
G10	Hs.8015	NM_012475	USP21	Ubiquitin specific peptidase 21	USP16/USP23	PPH07578A
G11	Hs.462492	NM_015276	USP22	Ubiquitin specific peptidase 22	USP3L	PPH07821A
G12	Hs.113876	NM_007331	NSD2	Nuclear receptor binding SET domain protein 2	KMT3F/KMT3G/MMSET/REIIBP/TRX5/WHS/WHSC1	PPH11148A
H01	Hs.520640	NM_001101	ACTB	Actin, beta	BRWS1/PS1TP5BP1	PPH00073G
H02	Hs.534255	NM_004048	B2M	Beta-2-microglobulin	-	PPH01094E
H03	Hs.592355	NM_002046	GAPDH	Glyceraldehyde-3-phosphate dehydrogenase	G3PD/GAPD/HEL-S-162eP	PPH00150F
H04	Hs.412707	NM_000194	HPRT1	Hypoxanthine phosphoribosyltransferase 1	HGPRT/HPRT	PPH01018C
H05	Hs.546285	NM_001002	RPLP0	Ribosomal protein, large, P0	L10E/LP0/P0/PRLP0/RPP0	PPH21138F
