# Supplementary material for: Epigenetic regulation in epithelial cells and innate lymphocyte responses to S. Typhi infection: insights into IFN-γ production and intestinal immunity
Source: Front Immunol. 2024 Sep 20;15:1448717. doi: 10.3389/fimmu.2024.1448717 (PMC11450450; doi:10.3389/fimmu.2024.1448717)
Supplement: Supplementary file 3 [file DataSheet3.pdf]

Supplemental Table 3

PCR Array Cat. #: PAHS-148Z **RT² Profiler™ PCR Array Human Antibacterial Response**

Position	Unigene	Refseq	Symbol	Description	Gname	RT2 Catalog
A01	Hs.525622	NM_005163	AKT1	V-akt murine thymoma viral oncogene homolog 1	AKT/CWS6/PKB/PKB-ALPHA/PRKBA/RAC/RAC-ALPHA	PPH00088B
A02	Hs.507080	NM_001639	APCS	Amyloid P component, serum	HEL-S-92n/PTX2/SAP	PPH07107A
A03	Hs.127799	NM_001165	BIRC3	Baculoviral IAP repeat containing 3	AIP1/API2/CIAP2/HAIP1/HIAP1/MALT2/MIHC/RNF49/c-IAP2	PPH00326B
A04	Hs.529019	NM_001725	BPI	Bactericidal/permeability-increasing protein	BPIFD1/rBPI	PPH16561B
A05	Hs.51120	NM_004345	CAMP	Cathelicidin antimicrobial peptide	CAP-18/CAP18/CRAMP/FALL-39/FALL39/HSD26/LL37	PPH09430A
A06	Hs.200242	NM_032587	CARD6	Caspase recruitment domain family, member 6	CINCIN1	PPH14265F
A07	Hs.694071	NM_052813	CARD9	Caspase recruitment domain family, member 9	CANDF2/hCARD9	PPH06127F
A08	Hs.2490	NM_033292	CASP1	Caspase 1, apoptosis-related cysteine peptidase	ICE/IL1BC/P45	PPH00105C
A09	Hs.599762	NM_001228	CASP8	Caspase 8, apoptosis-related cysteine peptidase	ALPS2B/CAP4/Casp-8/FLICE/MACH/MCH5	PPH00359F
A10	Hs.514107	NM_002983	CCL3	Chemokine (C-C motif) ligand 3	G0S19-1/LD78ALPHA/MIP-1-alpha/MIP1A/SCYA3	PPH00566F
A11	Hs.514821	NM_002985	CCL5	Chemokine (C-C motif) ligand 5	D17S136E/RANTES/SCYA5/SIS-delta/SISd/TCP228/eoCP	PPH00703B
A12	Hs.163867	NM_000591	CD14	CD14 molecule	-	PPH05723A
B01	Hs.198998	NM_001278	CHUK	Conserved helix-loop-helix ubiquitous kinase	IKBKA/IKK-alpha/IKK1/IKKA/NFKBIKA/TCF16	PPH00649C
B02	Hs.709456	NM_000567	CRP	C-reactive protein, pentraxin-related	PTX1	PPH02632A
B03	Hs.421724	NM_001911	CTSG	Cathepsin G	CATG/CG	PPH01358A
B04	Hs.789	NM_001511	CXCL1	Chemokine (C-X-C motif) ligand 1	FSP/GRO1/GROa/MGSA/MGSA-a/NAP-3/SCYB1	PPH00696C
B05	Hs.75765	NM_002089	CXCL2	Chemokine (C-X-C motif) ligand 2	CINC-2a/GRO2/GROb/MGSA-b/MIP-2a/MIP2/MIP2A/SCYB2	PPH00552F
B06	Hs.279611	NM_004406	DMBT1	Deleted in malignant brain tumors 1	GP340/muclin	PPH15755A
B07	Hs.86131	NM_003824	FADD	Fas (TNFRSF6)-associated via death domain	GIG3/MORT1	PPH00367A
B08	Hs.525600	NM_001017963	HSP90AA1	Heat shock protein 90kDa alpha (cytosolic), class A member 1	EL52/HSP86/HSP89A/HSP90A/HSP90N/HSPC1/HSPCA	PPH63391B
B09	Hs.37026	NM_024013	IFNA1	Interferon, alpha 1	IFL/IFN/IFN-ALPHA/IFN-alphaD/IFNA13/IFNA@	PPH01321B
B10	Hs.93177	NM_002176	IFNB1	Interferon, beta 1, fibroblast	IFB/IFF/IFN-beta/IFNB	PPH00384F
B11	Hs.597664	NM_001556	IKBKB	Inhibitor of kappa light polypeptide gene enhancer in B-cells	IKK-beta/IKK2/IKKB/IMD15/NFKBIKB	PPH00780C
B12	Hs.673	NM_000882	IL12A	Interleukin 12A	CLMF/IL-12A/NFSK/NKSF1/P35	PPH00544B
C01	Hs.674	NM_002187	IL12B	Interleukin 12B	CLMF/CLMF2/IL-12B/IMD28/IMD29/NKSF/NKSF2	PPH00545A
C02	Hs.83077	NM_001562	IL18	Interleukin 18 (interferon-gamma-inducing factor)	IGIF/IL-18/IL-1g/IL1F4	PPH00580C
C03	Hs.126256	NM_000576	IL1B	Interleukin 1, beta	IL-1/IL1-BETA/IL1F2	PPH00171C
C04	Hs.654458	NM_000600	IL6	Interleukin 6 (interferon, beta 2)	BSF2/HGF/HSF/IFNB2/IL-6	PPH00560C
C05	Hs.624	NM_000584	CXCL8	Interleukin 8	GCP-1/GCP1/IL8/LECT/LUCT/LYNAP/MDNCF/MONAP	PPH00568A
C06	Hs.522819	NM_001569	IRAK1	Interleukin-1 receptor-associated kinase 1	IRAK/pelle	PPH00835A
C07	Hs.369265	NM_007199	IRAK3	Interleukin-1 receptor-associated kinase 3	ASRT5/IRAKM	PPH06041A
C08	Hs.521181	NM_001098629	IRF5	Interferon regulatory factor 5	SLEB10	PPH02870A
C09	Hs.166120	NM_001572	IRF7	Interferon regulatory factor 7	IMD39/IRF-7H/IRF7A/IRF7B/IRF7C/IRF7H	PPH02014F
C10	Hs.696684	NM_002228	JUN	Jun proto-oncogene	AP-1/AP1/c-Jun	PPH00095A
C11	Hs.154078	NM_004139	LBP	Lipopolysaccharide binding protein	BPIFD2	PPH01424F
C12	Hs.204238	NM_005564	LCN2	Lipocalin 2	24p3/MSFI/NGAL/p25	PPH00446E
D01	Hs.529517	NM_002343	LTF	Lactotransferrin	GIG12/HEL110/HLF2/LF	PPH05751A
D02	Hs.726603	NM_015364	LY96	Lymphocyte antigen 96	ESOP-1/MD-2/MD2/ly-96	PPH06052A
D03	Hs.524579	NM_000239	LYZ	Lysozyme	LZM	PPH14748A
D04	Hs.145442	NM_002755	MAP2K1	Mitogen-activated protein kinase kinase 1	CFC3/MAPKK1/MEK1/MKK1/PRKMK1	PPH00711C
D05	Hs.514012	NM_002756	MAP2K3	Mitogen-activated protein kinase kinase 3	MAPKK3/MEK3/MKK3/PRKMK3/SAPKK-2/SAPKK2	PPH00747F
D06	Hs.514681	NM_003010	MAP2K4	Mitogen-activated protein kinase kinase 4	JNKK/JNKK1/MAPKK4/MEK4/MKK4/PRKMK4/SAPKK-1	PPH00195C
D07	Hs.594838	NM_003188	MAP3K7	Mitogen-activated protein kinase kinase kinase 7	MEKK7/TAK1/TGF1a	PPH00749C
D08	Hs.431850	NM_002745	MAPK1	Mitogen-activated protein kinase 1	ERK/ERK-2/ERK2/ERT1/MAPK2/P42MAPK/PRKM1/PRKM2	PPH00715B
D09	Hs.485233	NM_001315	MAPK14	Mitogen-activated protein kinase 14	CSBP/CSBP1/CSBP2/CSPB1/EXIP/Mxi2/PRKM14/PRKM15/RK	PPH00750B
D10	Hs.861	NM_002746	MAPK3	Mitogen-activated protein kinase 3	ERK-1/ERK1/ERT2/HS44KDAP/HUMKER1A/P44ERK1/P44MAPK	PPH00721F
D11	Hs.138211	NM_002750	MAPK8	Mitogen-activated protein kinase 8	JNK/JNK-46/JNK1/JNK1A2/JNK21B1/2/PRKM8/SAPK1/SAPK1c	PPH00720B
D12	Hs.632221	NM_000243	MEFV	Mediterranean fever	FMF/MEF/TRIM20	PPH13601A
E01	Hs.458272	NM_000250	MPO	Myeloperoxidase	-	PPH06082F
E02	Hs.82116	NM_002468	MYD88	Myeloid differentiation primary response gene (88)	MYD88D	PPH00911B
E03	Hs.646951	NM_004536	NAIP	NLR family, apoptosis inhibitory protein	BIRC1/NLRB1/psiNAIP	PPH00909E
E04	Hs.618430	NM_003998	NFKB1	Nuclear factor of kappa light polypeptide gene enhancer in B-cells 1	EBP-1/KBF1/NF-kB1/NF-kappa-B/NF-kappaB/NFKB-p105/NFKB-p50	PPH00204F
E05	Hs.81328	NM_020529	NFKBIA	Nuclear factor of kappa light polypeptide gene enhancer in B-cells inhibitor, alpha	IKBA/MAD-3/NFKBI	PPH00170F
E06	Hs.574741	NM_021209	NLRC4	NLR family, CARD domain containing 4	AIFEC/CARD12/CLAN/CLAN1/CLANA/CLANB/CLANC/CLAND	PPH06124A
E07	Hs.652273	NM_033004	NLRP1	NLR family, pyrin domain containing 1	CARD7/CIDED/CLR17.1/DEFCAP/DEFCAP-L/S/NAC/NALP1/PP1044	PPH06155E
E08	Hs.159483	NM_183395	NLRP3	NLR family, pyrin domain containing 3	AGTAVPRL/AII/AVP/C1orf7/CIAS1/CLR1.1/FCAS/FCAS1/FCU/MWS	PPH13170A
E09	Hs.738731	NM_006092	NOD1	Nucleotide-binding oligomerization domain containing 1	CARD4/CLR7.1/NLRC1	PPH00891C
E10	Hs.592072	NM_022162	NOD2	Nucleotide-binding oligomerization domain containing 2	ACUG/BLAU/CARD15/CD/CLR16.3/IBD1/NLRC2/NOD2B/PSORAS1	PPH06126F
E11	Hs.553498	NM_006218	PIK3CA	Phosphoinositide-3-kinase, catalytic, alpha polypeptide	CLOVE/CWS5/MCAP/MCM/MCMTC/PI3K/p110-alpha	PPH01355A
E12	Hs.928	NM_002777	PRTN3	Proteinase 3	ACPA/AGP7/C-ANCA/CANCA/MBN/MBT/NP-4/NP4/P29/PR-3/PR3	PPH07029A
F01	Hs.129758	NM_003978	PSTPIP1	Proline-serine-threonine phosphatase interacting protein 1	CD2BP1/CD2BP1L/CD2BP1S/H-PIP/PAPAS/PSTPIP	PPH11981A
F02	Hs.499094	NM_013258	PYCARD	PYD and CARD domain containing	ASC/CARD5/TMS/TMS-1/TMS1	PPH00907A
F03	Hs.413812	NM_006908	RAC1	Ras-related C3 botulinum toxin substrate 1	MIG5/Rac-1/TC-25/p21-Rac1	PPH00733F
F04	Hs.502875	NM_021975	RELA	V-rel reticuloendotheliosis viral oncogene homolog A (avian)	NFKB3/p65	PPH01812B
F05	Hs.519842	NM_003804	RIPK1	Receptor (TNFRSF)-interacting serine-threonine kinase 1	RIP/RIP1	PPH00335C
F06	Hs.103755	NM_003821	RIPK2	Receptor-interacting serine-threonine kinase 2	CARD3/CARDIAK/CCK/GIG30/RICK/RIP2	PPH00881C
F07	Hs.591607	NM_000578	SLC11A1	Solute carrier family 11 (proton-coupled divalent metal ion transporters)	LSH/NRAMP/NRAMP1	PPH05732F
F08	Hs.517070	NM_003064	SLPI	Secretory leukocyte peptidase inhibitor	ALK1/ALP/BLPI/HUSI/HUSI-I/MPI/WAP4/WFDC4	PPH02863A
F09	Hs.281902	NM_006704	SUGT1	SGT1, suppressor of G2 allele of SKP1 (S. cerevisiae)	SGT1	PPH05871A
F10	Hs.29344	NM_182919	TICAM1	Toll-like receptor adaptor molecule 1	IIAE6/MyD88-3/PRVTIRB/TICAM-1/TRIF	PPH06044A
F11	Hs.718838	NM_021649	TICAM2	Toll-like receptor adaptor molecule 2	MyD88-4/TICAM-2/TIRAP3/TIRP/TRAM	PPH06042A
F12	Hs.537126	NM_001039661	TIRAP	Toll-interleukin 1 receptor (TIR) domain containing adaptor protein	BACTS1/Mal/MyD88-2/wyatt	PPH06246B
G01	Hs.654532	NM_003263	TLR1	Toll-like receptor 1	CD281/TIL/TIL. LPRS5/rsc786	PPH01799A
G02	Hs.519033	NM_003264	TLR2	Toll-like receptor 2	CD282/TIL4	PPH01808A
G03	Hs.174312	NM_138554	TLR4	Toll-like receptor 4	ARMD10/CD284/TLR-4/TOLL	PPH01795F
G04	Hs.604542	NM_003268	TLR5	Toll-like receptor 5	MELIOS/SLE1/SLEB1/TIL3	PPH01793F
G05	Hs.575090	NM_006068	TLR6	Toll-like receptor 6	CD286	PPH01798E
G06	Hs.87968	NM_017442	TLR9	Toll-like receptor 9	CD289	PPH01809A
G07	Hs.241570	NM_000594	TNF	Tumor necrosis factor	DIF/TNF-alpha/TNFA/TNFSF2	PPH00341F
G08	Hs.713833	NM_001065	TNFRSF1A	Tumor necrosis factor receptor superfamily, member 1A	CD120a/FPF/MS5/TBP1/TNF-R/TNF-R-I/TNF-R55/TNFAR/TNFR1	PPH00346C
G09	Hs.368527	NM_019009	TOLLIP	Toll interacting protein	IL-1RAcPIP	PPH05844C
G10	Hs.591983	NM_004620	TRAF6	TNF receptor-associated factor 6	MGC:3310/RNF85	PPH00329B
G11	Hs.356076	NM_001167	XIAP	X-linked inhibitor of apoptosis	API3/BIRC4/IAP-3/ILP1/MIHA/XLP2/hIAP-3/hIAP3	PPH00323A
G12	Hs.302123	NM_030776	ZBP1	Z-DNA binding protein 1	C20orf183/DAI/DLM-1/DLM1	PPH16531C
H01	Hs.520640	NM_001101	ACTB	Actin, beta	BRWS1/PS1TP5BP1	PPH00073G
H02	Hs.534255	NM_004048	B2M	Beta-2-microglobulin	-	PPH01094E
H03	Hs.592355	NM_002046	GAPDH	Glyceraldehyde-3-phosphate dehydrogenase	G3PD/GAPD/HEL-S-162eP	PPH00150F
H04	Hs.412707	NM_000194	HPRT1	Hypoxanthine phosphoribosyltransferase 1	HGPRT/HPRT	PPH01018C
H05	Hs.546285	NM_001002	RPLP0	Ribosomal protein, large, P0	L10E/LP0/P0/PRLP0/RPP0	PPH21138F
